# Clinical Spectrum of Xeroderma Pigmentosum: An Observational Study

**DOI:** 10.7759/cureus.93993

**Published:** 2025-10-07

**Authors:** Usha Sri Akkineni, Dilip Chandra Chintada, Kirankanth Vudayana, Pooja Unnikrishnan

**Affiliations:** 1 Dermatology, Venereology and Leprosy, Great Eastern Medical School and Hospital, Srikakulam, IND

**Keywords:** dna repair, genetic counseling, malignancies, uv radiation, xeroderma pigmentosum

## Abstract

Background

Xeroderma pigmentosum (XP) is an uncommon condition caused by impaired UV radiation-induced damage repair. It is brought on by deficiencies in either post-replication repair or nucleotide excision repair, which can result in neurological, ophthalmic, and cutaneous problems. With a focus on mucocutaneous symptoms, associated malignancies, and systemic involvement, this study aims to record and examine the diverse clinical presentations of XP.

Methodology

A total of 10 clinically diagnosed XP patients participated in this 12-month prospective observational study conducted at a tertiary care center between April 2024 and April 2025. A comprehensive history, consent, and a dermatological, ophthalmic, neurological, and systemic examination were obtained.

Results

Of the 10 cases, there were four (40%) female and 6 (60%) male cases. The age of onset was less than one year in seven (70%) cases, between one and two years in two (20%) cases, and more than two years in one (10%) case. Poikiloderma (10, 100%), freckles (8, 80%), xerosis (8, 80%), lentigenes (6, 60%), skin atrophy (6, 60%), and seborrheic keratosis (4, 40%) were among the cutaneous findings. Neurological involvement was present in two (20%) cases. The ocular findings included photophobia in seven (70%) cases, conjunctival xerosis in six (60%) cases, and cataract in five (50%) cases. Further, two (20%) squamous cell carcinomas, three (30%) basal cell carcinomas, one (10%) lip carcinoma, one (10%) tongue carcinoma, and two (20%) buccal cavity carcinomas were among the mucocutaneous malignancies detected. The extracutaneous cancers observed were one (10%) sarcoma and one (10%) stomach carcinoma.

Conclusions

XP exhibits a wide spectrum of clinical manifestations, including aggressive cancers and pigmentary abnormalities. Enhancing life expectancy and quality of life requires early diagnosis, careful screening for cancer, sun protection, and genetic counseling.

## Introduction

Xeroderma pigmentosum (XP) is a rare autosomal recessive disease with 100% penetrance, caused by mutations in any of eight genes. The products of seven of these (XP-A through G) participate in the nucleotide excision repair mechanism, which fixes UV-induced photoproducts in DNA. Replication of DNA with unrepaired damage requires the eighth gene (XPV). It affects individuals across all racial groups and continents. Both men and women are equally impacted. It is characterized by a high sensitivity to sunlight, which can lead to sunburn, changes in skin pigmentation, and a significantly higher risk of developing skin cancer [1]. Skin atrophy, seborrheic keratosis, actinic keratosis, lentiginous pigmentation, photosensitivity, and seborrheic warts are common cutaneous features of the disease.

Ocular abnormalities, resulting from UV-induced DNA modification in conjunctival, corneal, and eyelid epithelial cells, are present in approximately 40% to 80% of patients with XP. Photophobia, severe keratitis, corneal opacification, and vascularization are its defining characteristics. About 20-30% of patients experience neurological issues, which can start anywhere from the age of two to middle life. These include spasticity, sensorineural deafness, convulsions, and intellectual disability [2].

Patients with XP are 1,000 times more likely to develop skin cancers in sun-exposed areas. The most prevalent kind is basal cell carcinoma (BCC), followed by malignant melanoma and squamous cell carcinoma (SCC). The incidence of other systemic cancers, such as breast, uterine, pancreatic, gastric, and mucosal carcinomas, is 10-20 times greater in XP [3].

Although the condition is ultimately fatal, lifespan can be extended by taking simple precautions to limit sun exposure. Extensive UV radiation-induced skin and eye damage in XP patients is indicative of a lack of awareness of the disease’s deadly nature, which leads to XP neglect [4]. With a focus on mucocutaneous symptoms, other features such as malignancies and systemic features have been described in this study.

## Materials and methods

Study design

A prospective, observational, pilot study was conducted over a 12-month period from April 2024 to April 2025 in the Department of Dermatology, after obtaining approval from the Institutional Ethics Committee, Great Eastern Medical School and Hospital (approval number: 09/IEC/GEMS&H/2025). The primary objective was to document and analyze the spectrum of clinical manifestations of XP, with a focus on mucocutaneous symptoms, related cancers, and systemic involvement.

Study population

The study included 10 consecutive patients with a confirmed clinical diagnosis of XP, irrespective of age and sex, who attended the dermatology outpatient clinic during the study period and were willing to provide consent. Patients with inadequate follow-up and those with photodermatoses or genodermatoses mimicking XP, where diagnosis could not be confirmed, were excluded.

Data collection

All patients underwent a detailed clinical evaluation and provided a comprehensive clinical history focusing on age of onset, gender, consanguinity, and symptom progression after providing written informed consent. Evaluation of pigmentation, freckling, skin malignancies, and atrophy was part of the dermatological examination. Relevant specialists performed neurological and ocular tests. Whenever feasible, dermoscopy and clinical imaging were employed. Laboratory investigations were performed to assess systemic involvement, and the data were compiled and tabulated.

Statistical analysis

Demographic characteristics, clinical presentations, and systemic findings were summarized descriptively. Data were analyzed using the OpenEpi software to provide a comprehensive overview of disease patterns within the study population.

## Results

Of the 10 patients included in the study, six (60%) were male and four (40%) were female. Seven cases (70%) had an age of onset before one year, followed by two (20%) cases between one and two years, and one (10%) case above two years. Consanguinity in marriage was found in four (40%) cases.

**Figure 1:**
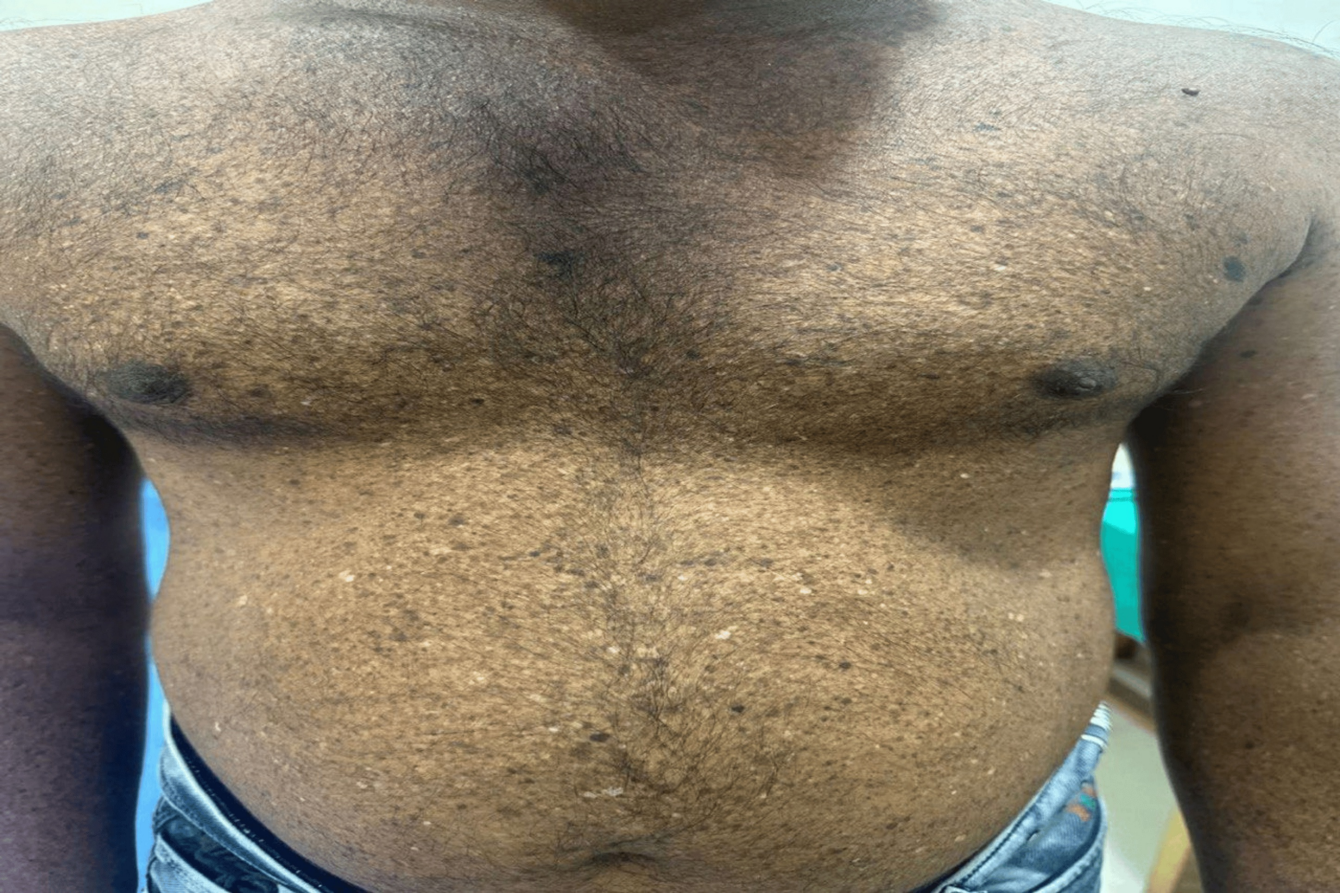
Clinical presentation of poikiloderma over the anterior trunk.

Skin abnormalities included poikiloderma (Figure 1) in 10 (100%) cases, freckles in eight (80%) cases, xerosis in eight (80%) cases, lentigenes in six (60%) cases, skin atrophy in six (60%) cases, seborrheic keratosis in four (40%) cases, nevi in three (30%) cases, and actinic keratosis in two (20%) cases.

**Figure 2:**
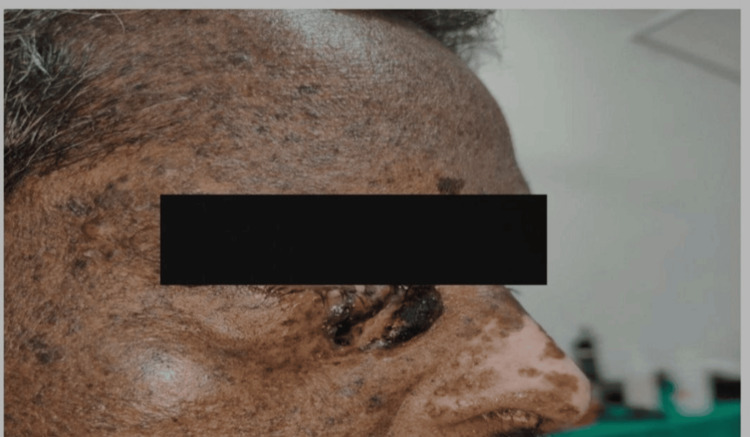
Clinical presentation of basal cell carcinoma near the right medial canthus.

**Figure 3:**
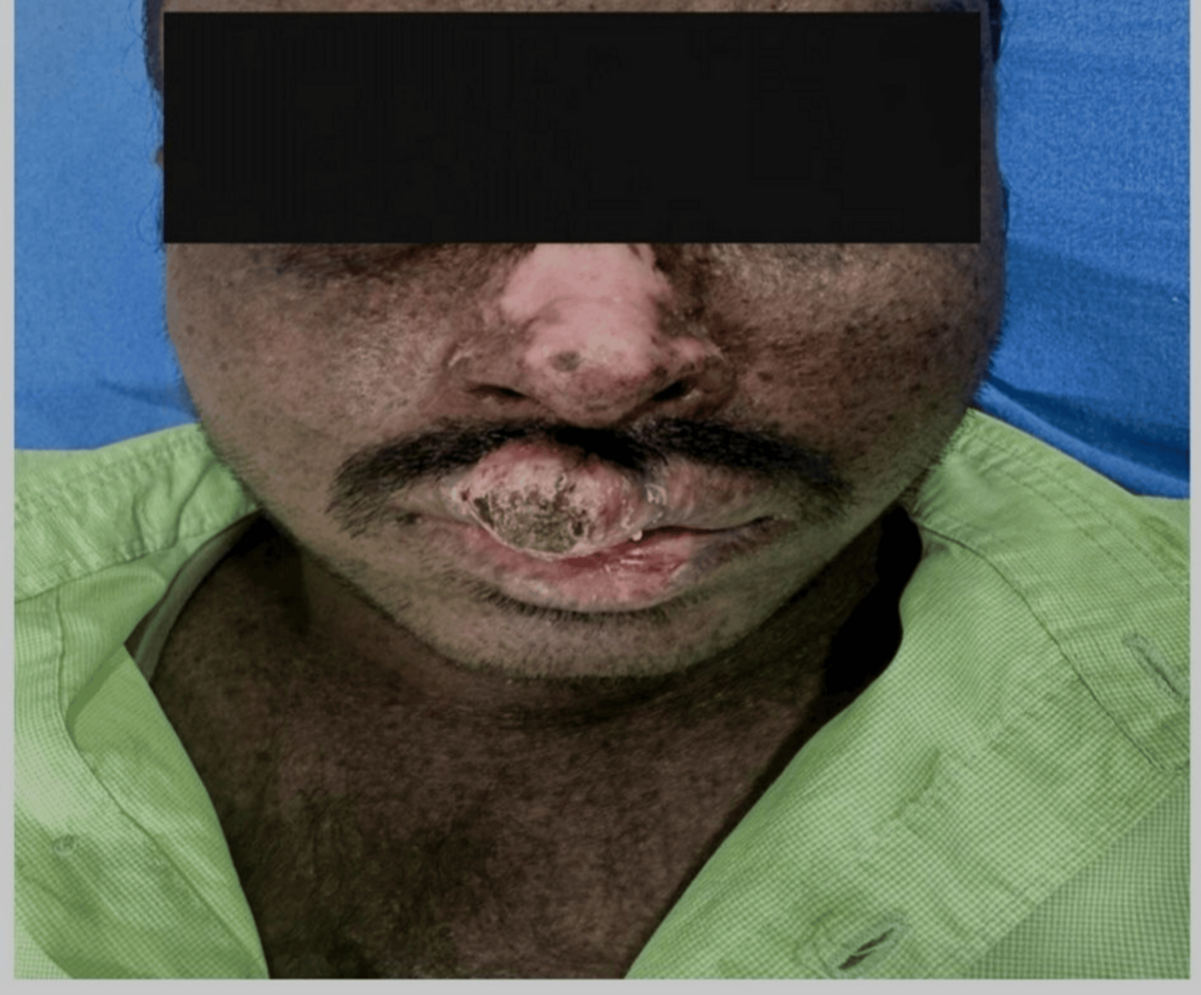
Clinical image of carcinoma of the upper lip in a patient with xeroderma pigmentosum.

**Figure 4:**
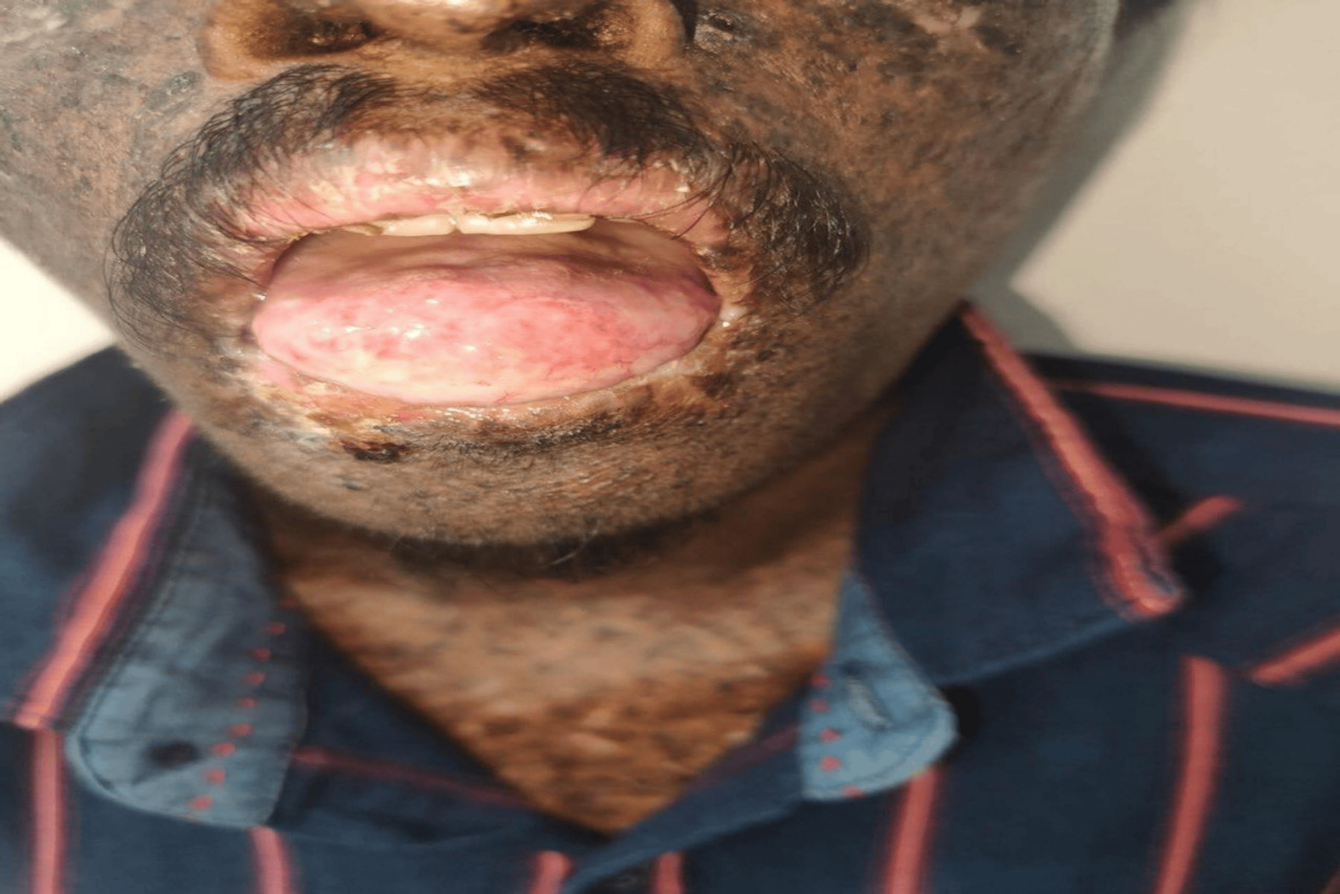
Clinical image of carcinoma of the tongue in a patient with xeroderma pigmentosum.

Overall, two (20%) cases out of 10 had neurological symptoms, such as delayed speech and abnormal gait. Further, seven (70%) cases had photophobia, six (60%) cases had conjunctival xerosis, and five (50%) cases had cataracts. However, among the mucocutaneous cancers, three (30%) cases had BCC (Figure 2), two (20%) cases had SCC, one (10%) case had eyelid carcinoma, one (10%) case had lip carcinoma (Figure 3), one (10%) cases had tongue carcinoma (Figure 4), and two (20%) cases had buccal cavity carcinoma (Table 1).

**Table 1:** Varied features of xeroderma pigmentosum.

Features	Clinical features	Number of cases	Percentage
Skin features	Poikiloderma	10	100
	Freckles	8	80
	Xerosis	8	80
	Lentigenes	6	60
	Skin atrophy	6	60
	Seborrheic keratosis	4	40
	Nevi	3	30
	Actinic keratosis	2	20
Ocular features	Photophobia	7	70
	Conjunctival xerosis	6	60
	Cataract	5	50
Neurological features	Speech delay, gait disturbances	2	20
Mucocutaneous malignancies and extracutaneous malignancies	Squamous cell carcinoma	2	20
	Basal cell carcinoma	3	30
	Lip carcinoma	1	10
	Tongue carcinoma	1	10
	Buccal cavity carcinoma	2	20
	Eyelid carcinoma	1	10
	Stomach carcinoma	1	10
	Sarcoma	1	10

**Figure 5:**
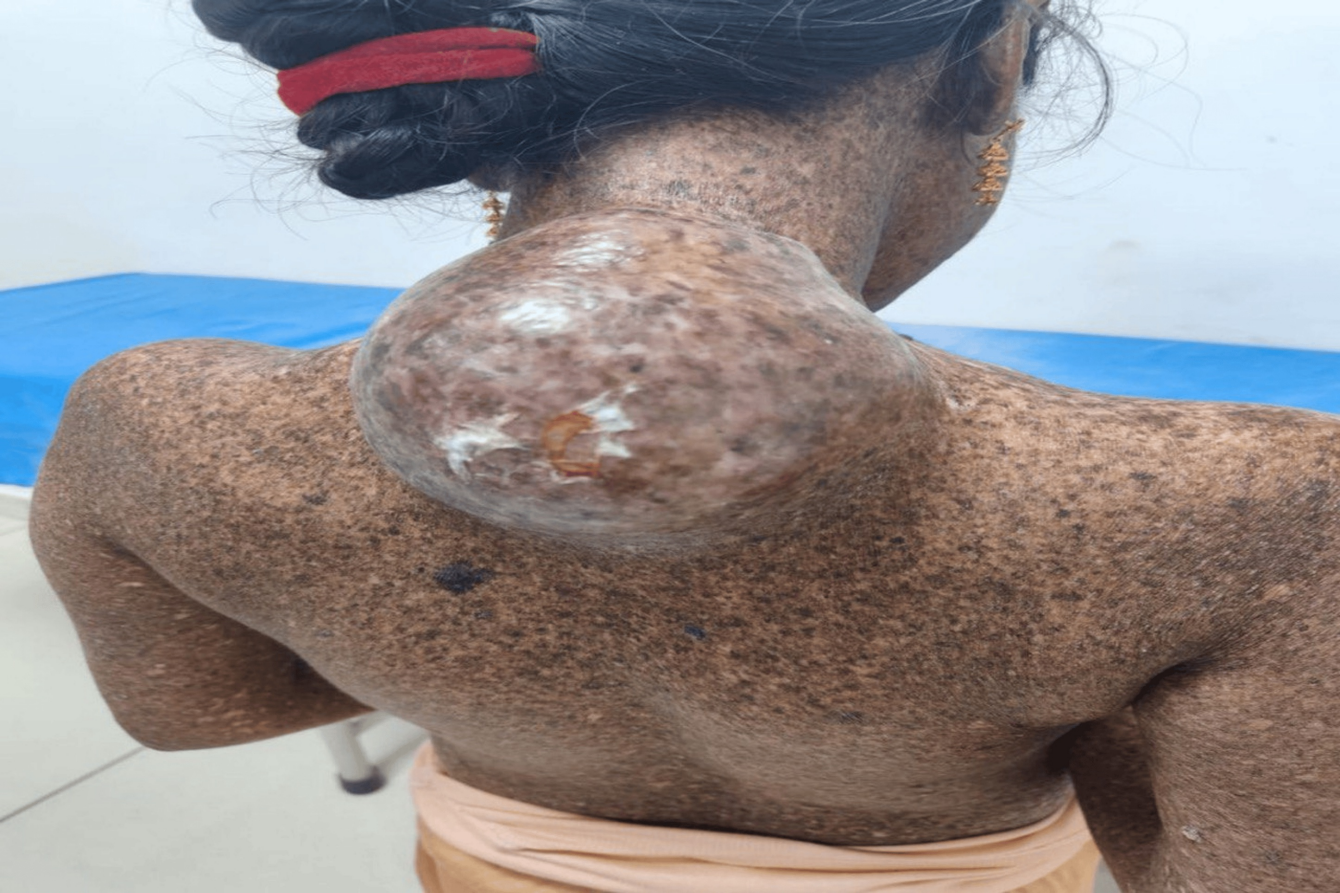
Clinical image showing sarcoma below the posterior aspect of the neck.

Among the extracutaneous cancers, one (10%) cases had sarcoma (Figure 5), and one (10%) case had stomach carcinoma.

## Discussion

This study included 10 clinically diagnosed XP cases, reinforcing the multisystem nature of the disease and highlighting uncommon findings such as gastrointestinal and mucosal cancers in Indian patients.

Demographic profile

Overall, seven (70%) cases in our analysis had a mean age of onset during the first year of life, which is consistent with findings by DiGiovanna and Kraemer (2012) [5], who found that cumulative UV exposure tends to cause initial skin alterations by the time a child is six months to three years old. Similar to the research by Halkud et al. (2014) [6], the male-to-female ratio (1.5:1) indicated no sex preference but rather a potentially greater detection rate among male youngsters because of increased solar exposure in rural areas.

Cutaneous manifestations

Poikiloderma, freckles, and xerosis were among the most common cutaneous symptoms detected in more than eight (80%) patients, which is comparable with the findings of Cleaver et al. (2009), who found these traits in 80-90% of cases [7].

Ocular manifestations

Ocular symptoms such as conjunctival xerosis, photophobia, and cataract were observed in seven (70%) patients. These findings are consistent with those reported by Brooks et al. (2013) [8], who found ocular involvement in more than two-thirds of XP patients, demonstrating that UV damage is not limited to the skin.

Neurological manifestations

Overall, two (20%) cases had neurological symptoms, such as cognitive impairment and speech delay. This is similar to the findings of Kraemer et al. (2003) [9] and Bradford et al. (2011) [10], who observed neurological symptoms in 20-30% of the XP-A and XP-D complementation groups. However, due to limited access to genetic typing, we were unable to classify our patients based on the complementation group.

Cutaneous malignancies

A notable characteristic of our study was the occurrence of cutaneous malignancies in five (50%) patients, including BCC in three (30%) and SCC in two (20%) cases, beginning as early as the second decade. This is consistent with the study reported by Aneja et al. (2025) [11], who found that XP patients have a >10,000-fold higher risk of non-melanoma skin cancer before the age of 20 than the general population.

Mucosal and internal malignancies

A notable characteristic of this series was the documentation of non-cutaneous cancers, which included mucosal malignancies such as lip, tongue, and buccal mucosa in a few individuals. Baskurt et al. (2024) previously described mucosal tumors in pediatric XP patients [12], notwithstanding their rarity. Additionally, gastric cancer was seen in one patient, an extremely unusual indication previously reported by Li et al. (2000) and hypothesized to result from worldwide DNA repair defects affecting the gastrointestinal mucosa [13]. Our findings highlight the importance of complete systemic surveillance in XP patients beyond dermatological follow-up. Our findings are consistent with previous XP literature regarding the frequency of cutaneous malignancies. However, the occurrence of mucosal and visceral tumors in two patients, while noteworthy, should be interpreted cautiously, given the small sample size and potential reporting bias.

Association with sarcoma

One (10%) patient had sarcoma, a finding that has been very rarely reported in the literature. While most likely coincidental, this observation may reflect a broader embryological disruption of developmental instability in XP patients and merits further assessment. Unlike many Western studies that utilize genetic profiling, our lack of complementation group typing limits the ability to establish direct genotype-phenotype correlations. However, the phenotypic diversity observed between patients reflects a varied genetic background, which may have been influenced by consanguinity, as seen in four (40%) cases. This is consistent with the research of Ijaz et al. (2019), who described a frequent trait in various Indian and Middle Eastern cohorts [14].

Management

Strict and consistent sun avoidance and protection and early detection and treatment of premalignant and malignant skin lesions are the mainstays of management [15]. Surgical removal of cancerous lesions and the use of antioxidants are considered to be effective against this disease [16]. Counselling on photoprotection, genetic counseling, and psychosocial support were given to all patients. Sun avoidance, UV-blocking clothing, and sunscreens were included in the management along with regular dermatological, ophthalmological, and neurological checkups.

Limitations

The lack of molecular or genetic subtyping, which would improve comprehension of phenotype-genotype connections, and the small sample size are the limitations of this study. Although malignancies were documented clinically, none of the cases had complete histologic confirmation. Standardized outcome measures and long-term follow-up were not feasible, which may limit the generalizability of the findings. Despite these constraints, the study provides valuable insight into the clinical variability and disease burden of XP in an underreported population.

## Conclusions

This study highlights the clinical spectrum of XP in an underreported population and suggests possible variation in malignancy patterns. For early detection and management of XP in India, our findings underscore the need for national guidelines, increased awareness, and vigilant clinical monitoring. Early genetic counseling can help families understand recurrence risk and enable informed reproductive choices, especially in populations with higher rates of consanguinity. Establishing multicenter registries and enabling wider access to genetic testing in the future can help improve outcomes.
